# Exercise intensity while hooked is associated with physiological status of longline-captured sharks

**DOI:** 10.1093/conphys/coy074

**Published:** 2018-12-20

**Authors:** Ian A Bouyoucos, Brendan S Talwar, Edward J Brooks, Jacob W Brownscombe, Steven J Cooke, Cory D Suski, John W Mandelman

**Affiliations:** 1 Shark Research and Conservation Program, Cape Eleuthera Institute, Rock Sound, The Bahamas; 2 Fish Ecology and Conservation Physiology Laboratory, Department of Biology and Institute of Environmental Science, Carleton University, Ottawa, ON, Canada; 3 Department of Natural Resources and Environmental Sciences, University of Illinois at Urbana-Champaign, Urbana, IL, USA; 4 Anderson Cabot Center for Ocean Life, New England Aquarium, Boston, MA, USA

**Keywords:** by-catch, elasmobranch, experimental fishing gear, fisheries, physiological stress, sub-lethal effect

## Abstract

Some shark populations face declines owing to targeted capture and by-catch in longline fisheries. Exercise intensity during longline capture and physiological status may be associated, which could inform management strategies aimed at reducing the impacts of longline capture on sharks. The purpose of this study was to characterize relationships between exercise intensity and physiological status of longline-captured nurse sharks (*Ginglymostoma cirratum*) and Caribbean reef sharks (*Carcharhinus perezi*). Exercise intensity of longline-captured sharks was quantified with digital cameras and accelerometers, which was paired with blood-based physiological metrics from samples obtained immediately post-capture. Exercise intensity was associated with physiological status following longline capture. For nurse sharks, blood pH increased with capture duration and the proportion of time exhibiting low-intensity exercise. Nurse sharks also had higher blood glucose and plasma potassium concentrations at higher sea surface temperatures. Associations between exercise intensity and physiological status for Caribbean reef sharks were equivocal; capture duration had a positive relation with blood lactate concentrations and a negative relationship with plasma chloride concentrations. Because Caribbean reef sharks did not appear able to influence blood pH through exercise intensity, this species was considered more vulnerable to physiological impairment. While both species appear quite resilient to longline capture, it remains to be determined if exercise intensity during capture is a useful tool for predicting mortality or tertiary sub-lethal consequences. Fisheries management should consider exercise during capture for sharks when developing techniques to avoid by-catch or reduce physiological stress associated with capture.

## Teaser

Fisheries management strategies are needed that improve the survival of shark by-catch in longline fisheries. We quantified exercise intensity and physiological stress of longline-caught sharks to understand why different individuals fare better or worse upon release. Nurse sharks could improve their condition with added rest, unlike Caribbean reef sharks.

## Introduction

Globally, some shark populations are in decline as a result of fisheries overexploitation and by-catch (Dulvy *et al.*, 2014). Longline fisheries are the predominant source of shark by-catch (Molina and Cooke, 2012; Oliver *et al.*, 2015) and can contribute to shark mortality owing to a suite of physiological perturbations that result from exhaustion during capture (Butcher *et al.*, 2015; Dapp *et al.*, 2016a). In addition to mortality, longline-caught sharks can experience numerous sub-lethal consequences associated with capture such as acid–base disruptions (Brooks *et al.*, 2012; Wilson *et al.*, 2014). Characterizing sub-lethal outcomes of capture is important because sub-lethal outcomes may result in population-level consequences, including reduced reproductive output (Skomal and Mandelman, 2012; Guida *et al.*, 2017b; Adams *et al.*, 2018). Defining sub-lethal outcomes of longline capture on sharks caught as by-catch is, therefore, of great value to fisheries management by informing strategies to mitigate stress.

Sharks exhibit species-specific physiological responses to longline capture (Gallagher *et al.*, 2014). This is well established in the literature; even congeneric species exhibit disparate physiological responses to longline capture (Mandelman and Skomal, 2009; Frick *et al.*, 2010; Marshall *et al.*, 2012; Butcher *et al.*, 2015). The mechanisms underlying inter-specific variability in the magnitude and intensity of the physiological response have not been characterized (although it may be related to aerobic metabolic scope), but this variability has been useful in identifying vulnerable species (Skomal and Bernal, 2010; Gallagher *et al.*, 2014). Sharks also exhibit intra-specific variability in physiological responses to capture, where differences in metrics have been generally attributed to capture duration, with mention of individuals’ respiratory physiology and struggling behaviour (Manire *et al.*, 2001; Jerome *et al.*, 2018). Laboratory evidence also demonstrates that acute physiological responses to a standardized experimental capture protocol are repeatable across days, suggesting that individuals exhibit unique physiological responses (Frick *et al.*, 2009). While physiological responses are cryptic and may be difficult to translate to conservation practices (Cooke and O’Connor, 2010), relating physiology to easily observable responses like exercise intensity or reflexes could produce valuable diagnostic tools for predicting an animal’s condition and informing species-specific management (Gallagher *et al.*, 2017; Jerome *et al.*, 2018).

Similar to physiological outcomes, the exercise intensity of sharks caught on longlines appears to be species-specific. Laboratory studies using experimental capture techniques have observed contrasts in exercise intensity during longline capture between species. Lemon sharks (*Negaprion brevirostris*) exposed to experimental longline capture did not rest and swam more than unhooked animals (Bouyoucos *et al.*, 2017). Port Jackson sharks (*Heterodontus portusjacksoni*) caught on experimental longlines rested following an initial bout of struggling (Frick *et al.*, 2010). Gummy sharks (*Mustelus antarcticus*) caught on longlines in the field rested throughout most of the capture event, whereas animals caught on experimental gear in the laboratory were nearly continuously active (Frick *et al.*, 2010; Guida *et al.*, 2016, 2017a). Intra-specific variation in responses to capture have received far less attention, although recent evidence of personality in sharks suggests that individuals exhibit unique, repeatable behaviours, including responses to capture and handling (Wilson *et al.*, 2015; Byrne*s et al.*, 2016a, 2016b; Finger *et al.*, 2018). Laboratory studies have been instrumental in providing preliminary insights into species-specific responses to capture, but there is a need for field-based studies to define intra- and inter-specific differences in exercise intensity of sharks during longline capture, especially given apparent contradictory responses between wild and captive studies.

Exercise intensity during longline capture may predict relative physiological status. Of the few studies that have quantified exercise metrics of captured sharks in various gear types, several have tested for associations between exercise intensity and physiological status at various points in time after the capture event. First, Frick *et al.* (2009) found no evidence of a relationship between peak whole-blood lactate concentrations and the amount of time Port Jackson sharks and Australian swellsharks (*Cephaloscyllium laticeps*) struggled in gill nets. Second, Guida *et al.* (2016) suggested that gummy sharks’ response of resting throughout demersal longline capture was responsible for the absence of an effect of capture duration on physiological status. These studies, however, do not support the idea that intra-specific variation in exercise intensity during capture is related to physiological status. Recently, Gallagher *et al.* (2017) provided evidence of an association between an acceleration-based metric of fight intensity and blood lactate concentrations across three shark species, although this trend likely reflects inter-specific variation, where nurse sharks (*Ginglymostoma cirratum*) generally had the lowest acceleration and blood lactate concentrations, and blacktip sharks (*Carcharhinus limbatus*) had the highest acceleration and blood lactate concentrations. In light of these three studies, there is a clear knowledge gap whether sharks’ exercise intensity during capture predicts physiological status.

The purpose of this study was to quantify why individual sharks fare better than others after longline capture. Our primary objective was to quantify associations between exercise intensity and physiological status of two shark species during longline capture, the nurse shark and Caribbean reef shark (*C. perezi*). Because behavioural (i.e. exercise intensity) and physiological responses of sharks appear to be repeatable at the level of individuals, we predicted that these species should exhibit intra-specific variation in exercise intensity that explains variation in physiological status. Our secondary objective was to compare associations between exercise intensity and physiological status between nurse sharks and Caribbean reef sharks. We predicted that relationships between exercise intensity and physiological status would differ in nature between the two study species because these species rely on different respiratory modes (stationary respiration and ram ventilation, respectively) that are generally associated with different levels of fisheries mortality among elasmobranchs (Dapp *et al.*, 2016b). Thus, our data have application for species-specific management, by identifying exercise intensity levels that predict ‘good’ relative physiological status, or a lack thereof that suggests a general species response. Finally, data on the nature of relationships between exercise intensity and physiological status can inform technical alterations to longline gear that serve to modify exercise intensity and reduce stress.

## Materials and methods

Ethical treatment of sharks was in accordance with permits MAF/FIS/17 and MAF/FIS/34 issued by the Bahamian Department of Marine Resources, and the permission to capture sharks within the Bahamian Shark Sanctuary was established in accordance with the Bahamian Department of Marine Resources Form 20 A, Regulation 36D (3), permitting fishing, possession and exportation of sharks or shark tissue. Animal care protocols were based on guidelines from the Association for the Study of Animal Behavior and the Animal Behavior Society (Rollin and Kessel, 1998).

### Experimental longline

**Figure 1:**
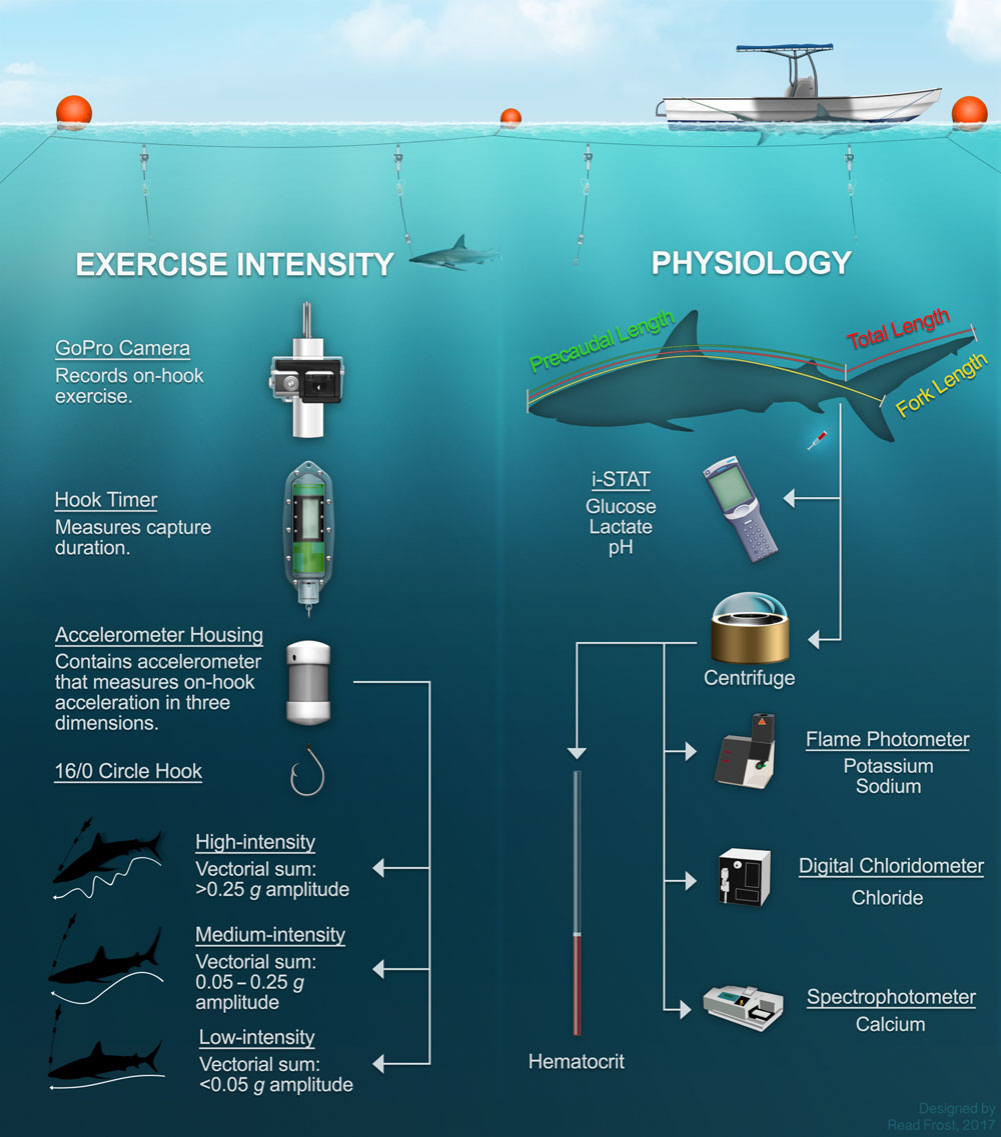
Diagram of experimental longline gear, *in situ* behavioural observation and assessment of physiological stress parameters.

Sharks were caught in coastal waters on experimental longlines (Fig. 1) between January 2012 and December 2013 around Cape Eleuthera, Eleuthera, The Bahamas (24.54° N, 76.12° W). Longlines were configured as mid-water sets based on the target species and habitat. Experimental longlines were 125.0 m long with six evenly spaced modified gangions. Each gangion was 1.3 m long and equipped with a digital camera (GoPro Hero 1 and 3 Silver, Woodman Labs Inc., Half Moon Bay, CA, USA), a hook timer (LP Hook Timer HT 600, Lindgren Pittman, Pompano Beach, FL, USA) and a tri-axial accelerometer (Hobo Pendant G Data Logger, Onset Computer Corporation, Bourne, MA, USA; 1 Hz recording frequency, ±3.0 *g* range, ±0.105 *g* accuracy, 0.025 *g* resolution) (*sensu*Grace *et al.*, 2010). Gangions consisted of a longline snap crimped to 0.15 m of monofilament attached to 1.0 m of braided polyester line, which was crimped onto 0.15 m of steel leader terminating in a 16/0 circle hook. Attachment points were conjoined with 8/0 swivels. Hook timers were rigged between monofilament and line sections, and accelerometers were set in a 7.6 × 3.8 cm polyvinylchloride capsule 15.0 cm above the circle hook on a steel leader. Longlines were checked every 30 min to assess the condition of sharks and monitor capture duration. Capture durations were limited to 4.5 h and intentionally manipulated to promote a broad range in capture durations (Brooks *et al.*, 2012). Before release, all sharks were restrained in the water alongside a boat for morphometric measurement, identification with dart tags and rototags, phlebotomy and hook removal. Sea surface temperatures (SSTs) were measured using a digital thermometer from mid-line at the beginning of each set.

### Assessment of physiological stress

A suite of secondary stress metrics were measured to characterize the acute physiological status of longline-captured sharks, including blood acid–base status (i.e. blood pH), lactate and glucose concentrations, haematocrit and plasma ion (sodium, potassium, chloride and calcium) concentrations. Blood was drawn from the caudal vasculature (i.e. *via* caudal puncture) using 38-mm 16 or 18 gauge needles and 3.0-ml syringes that were washed with sodium heparin. Following phlebotomy, whole blood was immediately transferred to an i-STAT CG4+ cartridge that was inserted into an i-STAT point-of-care device (Abbot Point of Care Inc., Princeton, NJ, USA) to measure blood pH and blood lactate concentration (Stoot *et al.*, 2014; Harter *et al.*, 2015). Because the i-STAT system measures blood pH at 37 °C (i.e. for use in homoeothermic animals), pH values were corrected to reflect SSTs—a proxy of body temperature—using species-independent conversion equations (Mandelman and Skomal, 2009; Brooks *et al.*, 2012). It should be noted, however, that the current ‘best-practice’ approach for measuring blood pH in elasmobranchs with the i-STAT system is by using the temperature correction function of the i-STAT itself rather than the species-independent conversion equations that were considered appropriate at the time of our study (Harter *et al.*, 2015). Blood was also transferred to an Accu-Chek portable blood glucose meter (Roche Diagnostics, Basel, Switzerland) to measure blood glucose concentration (Brooks *et al.*, 2012). Remaining blood was transferred to a vacutainer coated in lithium heparin and was stored on ice for 2 h until blood could be analysed for additional physiological stress metrics at a laboratory facility.

To measure haematocrit, a small volume of whole blood was transferred to a 75-mm micro-haematocrit tube and spun at 4400 *g* for 5 min in a micro-haematocrit centrifuge (Brooks *et al.*, 2012). Remaining whole blood was spun at 10000 *g* for 5 min, and plasma was aliquoted into 1.5-ml microcentrifuge tubes (Brooks *et al.*, 2012). Plasma samples were stored at −20°C prior to storage in liquid nitrogen and transport to an off-site laboratory, where samples were stored at −80°C. Plasma sodium and potassium were measured using a single-channel digital flame photometer (Model 2655-00, Cole Parmer, Vernon Hills, IL, USA), plasma chloride was quantified with a digital chloridometer (Model 4435000, Labconco Corporation, Kansas City, MO, USA), and plasma calcium was quantified using a commercially available kit (QuantiChrom Calcium Assay Kit, DICA-500, Bioassay Systems, Hayward, CA, USA) and analysed in a commercially available spectrophotometer (Spectra Max Plus 384, model 05362, Molecular Devices, Union City, CA, USA).

### Assessment of exercise intensity in hooked sharks

Acceleration data were analysed to distinguish exercise intensity levels during capture and generate acceleration-based metrics of exercise intensity. Raw acceleration data in each axis were converted to vectorial sums using HOBOware Graphing and Analysis Software (Onset Computer Corporation, Bourne, MA, USA). To generate accelerometric criteria to distinguish exercise intensity levels, time series of vectorial sum data ($\mathrm{VS}=\sqrt{x^{2}+y^{2}+z^{2}}$) were analysed using *k*-means clustering (Sakamoto *et al.*, 2009) to identify distinct exercise intensity levels for both species. Accelerometer-derived vectorial sum data were selected as a proxy for exercise intensity because variation in body acceleration is associated with metabolic energy expenditure (Gleis*s et al.*, 2011). Furthermore, vectorial sum is the preferred metric to overall dynamic body acceleration when the orientation of the data logger cannot be standardized across animals (e.g. hooking location), as was the case with gangions in this study (Qasem *et al.*, 2012).

Vectorial sum data from each individual were clustered across three groups. Characteristics (amplitude and cycle of the acceleration signal) of the three discrete clusters were visually inspected across all individuals to identify cutoff criteria for defining three exercise intensity levels: ‘high-intensity’ exercise had an amplitude > 0.25 *g* and a < 5-s cycle, ‘medium-intensity’ exercise had an amplitude < 0.25 *g* but > 0.05 *g*, or an amplitude > 0.25 *g* with a > 5-s cycle, and ‘low-intensity’ exercise referred to any signal with an amplitude < 0.05 *g*. After defining cutoff criteria, individuals’ clusters were each assigned an exercise intensity level as detailed above. Therefore, although acceleration data from all animals were clustered into three groups, not all animals exhibited all three exercise intensity levels. As such, this approach accounted for any biases that would occur from *k*-means clustering across individuals. Analysis of video data corroborated acceleration data: high-intensity exercise referred to burst swimming and/or strong contortions, medium-intensity exercise referred to brief aggravated movement, and slow directional or circular swimming and low-intensity exercise referred to sharks resting on the bottom or suspended vertically on a gangion.

### Data analysis

**Figure 2:**
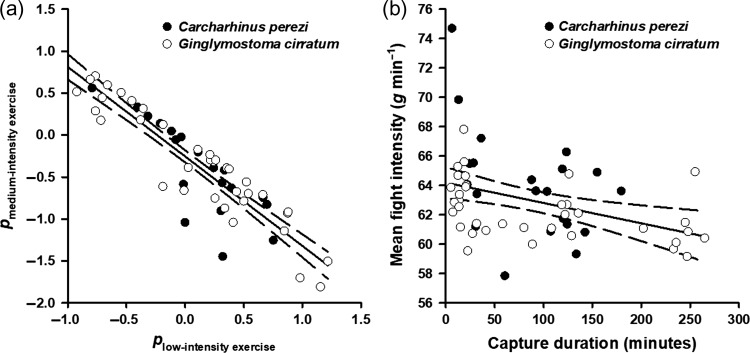
Correlations between possible explanatory exercise metrics. Proportions (**a**) of the total capture event when sharks exhibited low- (*p*_low-intensity exercise_) and medium-intensity (*p*_medium-intensity exercise_) exercises were correlated (Linear regression, *R*^2^ = 0.84, *F*_1, 55_ = 290.96, *P* < 0.001) and are presented as log-ratio transformed proportions. Mean fight intensity (**b**; in *g* min^−1^ where *g* = 9.81 m s^−2^) is measured as the sum of acceleration values recorded during a capture event divided by capture duration and had a negative linear relationship with capture duration (Linear regression, *R*^2^ = 0.14, *F*_1, 55_ = 9.54, *P* = 0.003). Data are pooled for nurse sharks (*Ginglymostoma cirratum*) and Caribbean reef sharks (*Carcharhinus perezi*).

One exercise metric was generated from accelerometric data: the proportion of the capture event that sharks exhibited low-intensity exercise (*p*_low_). Proportion data were log-ratio transformed to account for the fact that raw proportions sum to one and are, therefore, not independent (Aebischer *et al.*, 1993). Including metrics for the proportion of time sharks exhibited medium- (*p*_med_) and high-intensity (*p*_high_) exercise was considered, but data exploration indicated that *p*_low_ and *p*_med_ were highly correlated (Fig. 2a), and variation in *p*_high_ would logically be mirrored by variation in *p*_low_, despite these transformed variables not being correlated. Other exercise metrics were considered (e.g. the frequency of bouts of high-intensity exercise or average fight intensity), but these metrics have previously been demonstrated to not be associated with stress markers (lactate, glucose and pH) and were both correlated with capture duration in this study (Fig. 2b) and elsewhere (Brownscombe *et al.*, 2014; Gallagher *et al.*, 2017). Notably, capture duration is often associated with stress markers for sharks (Skomal, 2006; Danylchuk *et al.*, 2014; Dapp *et al.*, 2016a; Whitney *et al.*, 2017). Therefore, capture duration was chosen as a proxy of sharks’ fight intensity, where longer capture durations were associated with lower average fight intensity (Brownscombe *et al.*, 2014). Capture duration was also an appropriate metric because it was not correlated with *p*_low_.

Linear models were used to investigate relationships between exercise metrics and physiological metrics. Separate models were analysed for each species in anticipation of species-specific stress responses (Mandelman and Skomal, 2009). Physiological metrics (plasma calcium, plasma sodium, plasma potassium, plasma chloride, blood glucose, blood lactate, blood pH and haematocrit) were fit as response variables with *p*_low_, capture duration, SST and total length (TL, in cm) as covariates. Statistical significance was determined by generating 95% confidence intervals of effect size from 1000 posterior simulations of factors in our linear models (Nakagawa and Cuthill, 2007). A factor was considered to be significant if the 95% confidence interval did not overlap zero (Hasle*r et al.*, 2016). Models were validated with Q–Q plots of model residuals to assess normality and plotting fitted/predicted values against model residuals to assess homogeneity of variances. All analyses were conducted in R using the R Stats Package (R Core Team, 2018). Posterior simulations were generated using the ‘arm’ package (Gelman and Su, 2018).

## Results

**Figure 3:**
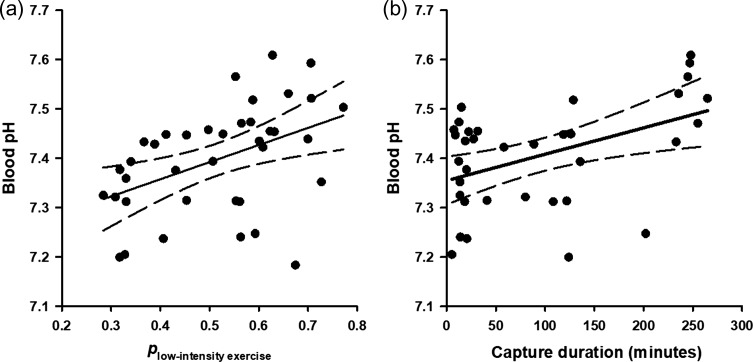
Relationship between exercise intensity (**a**) and capture duration (**b**) of longline-caught nurse sharks (*Ginglymostoma cirratum*) and physiological status. The proportion of time sharks exhibited low-intensity exercise (*p*_low-intensity__exercise_) refers to periods of inactivity or resting. Proportion data were log-ratio transformed for analyses and were back-transformed for presentation. Dashed lines represent 95% confidence intervals.

**Figure 4:**
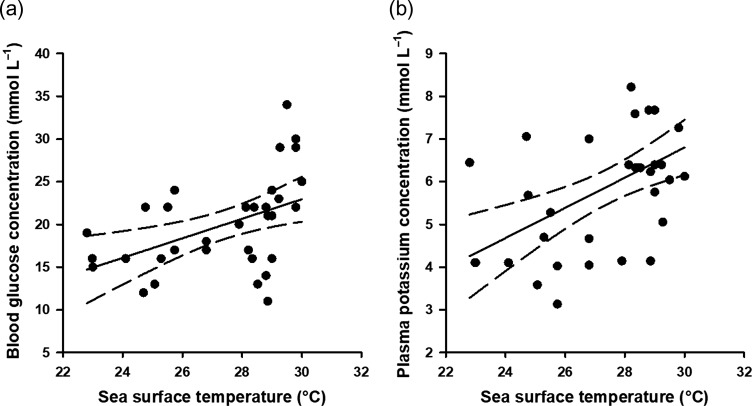
Relationships between sea surface temperature and physiological status of longline-caught nurse sharks (*Ginglymostoma cirratum*). Glucose concentrations (**a**) were measured from whole blood, and potassium concentrations (**b**) were measured from plasma. Dashed lines represent 95% confidence intervals.

**Figure 5:**
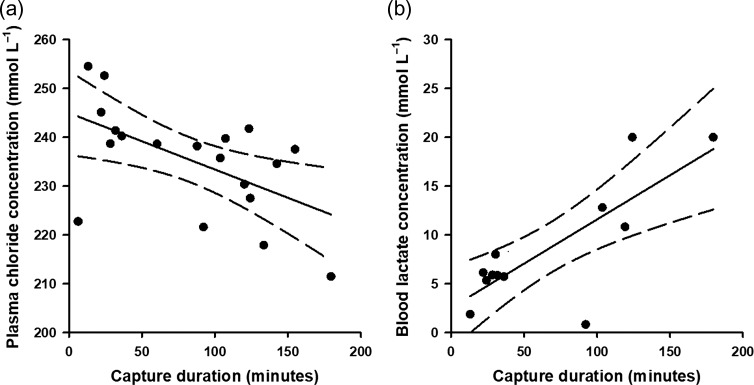
Relationships between capture duration of longline-caught Caribbean reef sharks (*Carcharhinus perezi*) and physiological status. Chloride concentrations (**a**) were measured from plasma, and lactate concentrations (**b**) were measured from whole blood. Dashed lines represent 95% confidence intervals.

Between 15 June 2012 and 13 January 2014, 36 Caribbean reef sharks and 44 nurse sharks were captured on experimental longlines. Physiological data, *p*_low_, capture duration, SST and TL of all sharks in this study are summarized in Table 1. Capture durations varied from 2.57 to 264.88 min. Variations in physiological stress metrics were associated with variation in exercise metrics for nurse sharks and Caribbean reef sharks (Table 2). For nurse sharks, blood pH increased with *p*_low_ and capture duration (Fig. 3). Blood glucose and plasma potassium concentrations increased with SST (Fig. 4), and plasma potassium concentrations decreased with increasing TL. For Caribbean reef sharks, plasma chloride concentrations decreased with capture duration, and blood lactate concentrations increased with capture duration (Fig. 5).

**Table 1:** Descriptive statistics of response variables (blood-based physiological data) and explanatory variables for nurse sharks (*Ginglymostoma cirratum*) and Caribbean reef sharks (*Carcharhinus perezi*). The proportion of time sharks exhibited low-intensity exercise (*p*_low_) refers to periods of inactivity or resting.

Species	Metric	*n*	Mean	Maximum	Minimum	S.D.
Nurse shark	Calcium (mmol l^−1^)	36	4.91	7.64	3.56	0.98
	Chloride (mmol l^−1^)	36	247.41	269.28	193.52	16.43
	Sodium (mmol l^−1^)	36	255.94	337.47	167.77	35.13
	Potassium (mmol l^−1^)	36	5.88	8.21	3.13	1.42
	Glucose (mmol l^−1^)	34	19.97	34.00	11.00	5.41
	pH	35	7.41	7.61	7.19	0.11
	Lactate (mmol l^−1^)	35	1.13	3.25	0.30	0.82
	Haematocrit (%)	34	17.42	29.03	7.14	4.41
	*p*_low_ (%)	42	52.87	77.16	28.43	14.24
	Capture duration (min)	42	95.68	264.88	5.33	91.96
	SST (°C)	41	27.51	30.00	22.80	2.14
	TL (cm)	44	206.68	260.00	125.00	27.06
Caribbean reef shark	Calcium (mmol l^−1^)	19	5.51	7.66	3.67	1.07
	Chloride (mmol l^−1^)	19	235.29	254.52	211.50	11.11
	Sodium (mmol l^−1^)	18	269.67	350.16	216.38	39.29
	Potassium (mmol l^−1^)	19	6.34	9.69	4.84	1.44
	Glucose (mmol l^−1^)	17	10.39	16.7	5.1	3.43
	pH	11	7.30	7.69	6.96	0.18
	Lactate (mmol l^−1^)	12	8.62	20.00	0.84	6.24
	Haematocrit (%)	20	20.32	27.90	9.59	5.74
	*p*_low_ (%)	32	50.49	73.96	24.02	12.42
	Capture duration (min)	32	76.22	179.58	2.57	50.12
	SST (°C)	28	26.58	31.60	23.50	2.01
	TL (cm)	36	148.97	211.00	82.10	31.34

**Table 2:** Linear model outputs (95% confidence interval limits) for the effect of exercise intensity (*p*_low_), capture duration, SST and TL on physiological stress parameters for nurse sharks (*Ginglymostoma cirratum*) and Caribbean reef sharks (*Carcharhinus perezi*). Bolded factors denote statistical significance, where the 95% confidence interval does not include zero.

	Nurse sharks			Caribbean reef sharks		
Response	Parameter	2.5%	97.5%	Parameter	2.5%	97.5%
Ca^2+^	**Intercept**	**1.09**	**13.04**	Intercept	−5.85	13.15
	*p*_low_	−0.69	0.75	*p*_low_	−1.59	0.95
	Capture duration	−0.00	0.01	Capture duration	−0.01	0.01
	SST	−0.31	0.08	SST	−0.25	0.47
	TL	−0.01	0.02	TL	−0.03	0.05
Na^+^	**Intercept**	**60.98**	**481.61**	Intercept	−107.39	536.02
	*p*_low_	−28.83	21.03	*p*_low_	−52.56	53.09
	Capture duration	−0.29	0.02	Capture duration	−0.28	0.67
	SST	−9.20	4.77	SST	−13.71	13.63
	TL	−0.16	0.79	TL	−0.92	1.26
K^+^	Intercept	−12.19	1.85	Intercept	−12.79	8.64
	*p*_low_	−0.35	1.28	*p*_low_	−0.57	2.15
	Capture duration	−0.00	0.01	Capture duration	−0.00	0.02
	**SST**	**0.30**	**0.76**	SST	−0.21	0.64
	**TL**	**−0.03**	**−0.00**	TL	−0.01	0.03
Cl^-^	**Intercept**	**126.52**	**307.95**	**Intercept**	**136.66**	**313.34**
	*p*_low_	−19.22	2.60	*p*_low_	−11.16	9.15
	Capture duration	−0.09	0.02	**Capture duration**	**−0.23**	**−0.02**
	SST	−0.92	4.81	SST	−2.72	4.02
	TL	−0.28	0.09	TL	−0.18	0.25
Glucose	Intercept	−52.21	10.39	Intercept	−269.66	147.68
	*p*_low_	−4.08	3.43	*p*_low_	−13.96	68.69
	Capture duration	−0.01	0.03	Capture duration	−0.04	0.58
	**SST**	**0.22**	**2.31**	SST	−5.82	8.94
	TL	−0.04	0.09	TL	−0.02	1.21
pH	**Intercept**	**7.10**	**8.19**	**Intercept**	**5.75**	**10.03**
	*p*_**low**_	**0.01**	**0.12**	*p*_low_	−0.55	0.48
	**Capture duration**	**0.00**	**0.00**	Capture duration	−0.00	0.00
	SST	−0.02	0.01	SST	−0.09	0.05
	TL	−0.00	0.00	TL	−0.01	0.01
Lactate	Intercept	−4.39	6.07	Intercept	−45.69	42.64
	*p*_low_	−1.01	0.26	*p*_low_	−10.20	5.84
	Capture duration	−0.01	0.00	**Capture duration**	**0.01**	**0.14**
	SST	−0.21	0.13	SST	−0.88	1.91
	TL	−0.01	0.02	TL	−0.21	0.09
Haematocrit	Intercept	−28.23	31.43	Intercept	−50.34	25.05
	*p*_low_	−4.19	2.56	*p*_low_	−7.45	5.16
	Capture duration	−0.02	0.02	Capture duration	−0.02	0.08
	SST	−0.44	1.37	SST	−0.43	2.40
	TL	−0.05	0.09	TL	−0.08	0.15

## Discussion

This study sought to better quantify the relationship between the exercise intensity of sharks while hooked on longlines and their physiological status upon landing. We found that nurse sharks that exhibited low-intensity exercise more frequently had higher blood pH relative to more active individuals, and blood pH increased with capture duration. Nurse sharks are a mostly sedentary species, possibly owing to this species’ low metabolic rates, and it follows that nurse sharks have been documented to rest on the substrate throughout a capture event (Whitney *et al.*, 2016; Gallagher *et al.*, 2017). Previously, it has been demonstrated that the number of burst swimming events during exercise has a positive linear relationship with the energetic cost of resolving physiological disruptions (e.g. acidosis) (Svendsen *et al.*, 2010). During intense activity in elasmobranchs, blood pH drops because of a combination of metabolic acidosis (release of metabolic protons from the white muscle following anaerobic lactate production) and respiratory acidosis (dissociation of protons and bicarbonate following carbon dioxide accumulation) (Wood *et al.*, 1983; Mandelman and Skomal, 2009). For nurse sharks, it is possible that fewer burst events during capture (i.e. a higher *p*_low_) resulted in a smaller magnitude of physiological disturbance (i.e. less pH decline or higher pH). Given capture duration’s inverse relationship with mean fight intensity, it is likely that more anaerobically fuelled struggling (i.e. high mean fight intensity or more high-intensity exercise) is associated with low blood pH. Furthermore, this relationship suggests that struggling occurs early in the capture event, with less intense activity occurring over longer durations. Alternatively, if fight intensity is not related to blood pH, nurse sharks could be recovering over long durations of longline capture by resting (Brooks *et al.*, 2012; Brownscombe *et al.*, 2014; Gallagher *et al.*, 2017). Gummy sharks were documented to remain stationary for ~90% of a longline capture event, such that blood pH (among other metrics) was not influenced by capture duration (Guida *et al.*, 2016). The presence or absence of an effect of capture duration on blood pH in two sharks that rest during capture could be related to differences in metabolic rates; nurse sharks are estimated to have lower metabolic rates than gummy sharks at comparable temperatures (Skomal and Mandelman, 2012; Morash *et al.*, 2016; Whitney *et al.*, 2016). Therefore, nurse sharks appear to be quite resilient to stress.

The physiological status of nurse sharks was also associated with changes in water temperatures. Nurse sharks caught at higher water temperatures had higher blood glucose and plasma potassium concentrations than animals captured at lower water temperatures. Positive relationships between SST and blood glucose have previously been reported for Atlantic sharpnose (*Rhizoprionodon terraenovae*), gummy and blacktip reef sharks (*C. melanopterus*) (Hoffmayer *et al.*, 2012; Guida *et al.*, 2016; Bouyoucos *et al.*, 2018). Variation in water temperature ranging from 23.0 to 30.0°C has previously been demonstrated to influence metabolic rate in nurse sharks and increases in blood glucose concentrations at higher temperatures may reflect increased energetic demand (as an oxidative fuel source), or increased rates of anaerobic glycolysis if an increased standard metabolic rate reduces the available aerobic scope (Whitney *et al.*, 2016). This study found evidence of a positive relationship between water temperatures and plasma potassium concentrations following capture, while a negative relationship has been reported elsewhere (Guida *et al.*, 2016). Contrasting trends in plasma electrolytes appear to be common in studies of elasmobranch stress (Skomal and Mandelman, 2012). High plasma potassium concentrations are generally associated with muscle tetany and, eventually, mortality in elasmobranchs (Moyes *et al.*, 2006; Skomal and Mandelman, 2012). Indeed, studies have reported tetany or poor subjective condition when plasma potassium concentrations exceeded the threshold for hyperkalemia (7.0 mmol l^−1^) in gummy (>7.0 mmol l^−1^), sandbar (*C. plumbeus*; >8.7 mmol l^−1^) and dusky sharks (*C. obscurus*; >9.3 mmol l^−1^) (Frick *et al.*, 2010; Butcher *et al.*, 2015). Sharks in our study generally had plasma potassium concentrations below 7.0 mmol l^−1^ (mean = 5.8 ± 1.4 mmol l^−1^), and sharks with plasma potassium concentrations eclipsing 7.0 mmol l^−1^ were typically caught at over 28°C. Thus, while it is possible that nurse sharks may be more vulnerable to experiencing hyperkalemia at higher water temperatures, this claim warrants additional research.

Associations between exercise intensity and physiological status were equivocal for Caribbean reef sharks. Blood lactate concentrations increased with capture duration, while capture duration correlated negatively with plasma chloride concentration. Average fight intensity and the frequency of high-intensity exercise decrease with increasing capture duration because high-intensity exercise (e.g. peak acceleration values) typically occurs only during the first few minutes of hook-and-line capture (Frick *et al.*, 2010; Brownscombe *et al.*, 2014; Guida *et al.*, 2016, 2017a; Gallagher *et al.*, 2017). Furthermore, exhaustive chasing protocols implemented in laboratory settings to simulate fishing capture last only several minutes and result in increasing blood lactate and decreasing pH over several hours (Brooks *et al.*, 2011a; Bouyoucos *et al.*, 2017). Together, these data suggest that longer capture durations allow for appreciable amounts of time for lactate to leak from the muscle into the blood, thereby making it possible to record high lactate concentrations despite sharks having low mean fight intensity values (Brooks *et al.*, 2011a; Hoffmayer *et al.*, 2015). Alternatively, these data also suggest that continued activity over longer capture durations supports continued lactate production as appears to be the case for rod-and-reel capture (French *et al.*, 2015; Whitney *et al.*, 2017). We could not replicate an effect of capture duration on blood pH for Caribbean reef sharks as we accomplished for blood lactate concentration, although this is likely the result of low sample sizes owing to high CG4+ cartridge failure rates (~60% failure rate) of the i-STAT system (Brooks *et al.*, 2012; Harter *et al.*, 2015). Overall, Caribbean reef sharks appear to exhibit consistent exercise intensity levels during longline capture, and that other extrinsic factors (e.g. environmental conditions) may be important for influencing physiological status.

In this study, Caribbean reef sharks also exhibited lower plasma chloride concentrations as capture duration increased. Effects of capture duration on plasma chloride concentrations are equivocal for elasmobranchs (Skomal and Mandelman, 2012); studies have documented no variation in plasma chloride concentrations with capture duration in bronze whaler (*C. brachyurus*), dusky and Caribbean reef sharks (Cliff and Thurman, 1984; Brooks *et al.*, 2012; Dapp *et al.*, 2016a), while others have reported negative relationships for dusky and sandbar sharks (Butcher *et al.*, 2015). Decreases in plasma chloride concentrations with increasing capture duration could be indicative of recovery over long capture durations (Brooks *et al.*, 2012). Alternatively, changes in plasma chloride concentrations can be explained in the context of capture duration’s inverse relationship with fight intensity (Brownscombe *et al.*, 2014). It is possible that plasma chloride concentrations increased with mean fight intensity because of increased anaerobic activity resulting in an acidosis that drives chloride out of the white muscle cells and into the plasma (Skomal and Mandelman, 2012). Additional research is warranted into the utility of plasma electrolytes as valuable stress markers for elasmobranchs.

Caribbean reef sharks appear to be more vulnerable to physiological impairment from longline capture than nurse sharks. Specifically, Caribbean reef sharks, unlike nurse sharks, did not exhibit associations between exercise metrics and blood pH. Sufficient declines in blood pH can ultimately be responsible for exercise-induced mortality of fishes (Wood *et al.*, 1983; Skomal and Mandelman, 2012), although this was not the case in our study, as we observed no at-vessel mortalities. Contrasting exercise intensity levels appear related to species’ general activity levels, ventilation strategies and metabolic rates, which influence species’ physiological status following fisheries interactions (Dapp *et al.*, 2016b; Gallagher *et al.*, 2017). Caribbean reef sharks likely exhibit metabolic rates characteristic of other sub-tropical carcharhinid sharks (e.g. lemon sharks or blacktip sharks), which are higher than for nurse sharks at comparable temperatures (Lear *et al.*, 2017). Caribbean reef sharks can buccal pump like nurse sharks (observed by gangion cameras), but their probable high metabolic rates likely require ram ventilation for effective gas exchange during and after exercise (Brooks *et al.*, 2011a). It should be noted that while we suggest Caribbean reef sharks are more vulnerable to changes in physiological status owing to longline capture, both species had 100% at-vessel survival and are generally considered to be physiologically resilient to capture (Brooks *et al.*, 2012, 2013; Gallagher *et al.*, 2017; Jerome *et al.*, 2018). In addition, other studies have documented very high post-release survival estimates following longline capture for Caribbean reef sharks (Brooks *et al.*, 2011b; Shipley *et al.*, 2017). We are unaware, however, of the potential for Caribbean reef sharks or nurse sharks to experience negative long-term sub-lethal consequences (i.e. a tertiary stress response) from longline capture, and the extent to which exercise intensity could influence outcomes.

In conclusion, our results suggest that sharks’ exercise intensity during longline capture plays an influential role in affecting physiological status upon release. However, for the two species studied here, their apparent physiological resilience to short durations of longline capture suggests that such activities are not overly detrimental (i.e. no at-vessel mortality). It is possible that the negative consequences of capture could be magnified given the context specificity of fisheries interactions with other gear type or configurations (e.g. hook type, gangion length) or deployment conditions (e.g. longer set times, presence of predators, water quality) (Raby *et al.*, 2015). Based on our data, strategies that minimize capture duration (gear that is easily depredated by sharks) or allow for sharks to engage in low-intensity exercise during capture may improve physiological status upon release. As our data also demonstrate, exercise intensity can be determined during capture as a meaningful predictor of physiological status upon release, although future research is warranted to establish whether exercise intensity is a useful predictor of post-release outcomes (e.g. mortality, recovery or tertiary sub-lethal responses). Ultimately, studies aimed at elasmobranch conservation will require a multi-disciplinary approach, including the integration of exercise and behaviour with physiology (see Cooke *et al.*, 2014).
